# Cerebrospinal Fluid BACE1 Activity and Brain Amyloid Load in Alzheimer's Disease

**DOI:** 10.1100/2012/712048

**Published:** 2012-01-03

**Authors:** Timo Grimmer, Panagiotis Alexopoulos, Amalia Tsolakidou, Liang-Hao Guo, Gjermund Henriksen, Behrooz H. Yousefi, Hans Förstl, Christian Sorg, Alexander Kurz, Alexander Drzezga, Robert Perneczky

**Affiliations:** ^1^Department of Psychiatry and Psychotherapy, Klinikum rechts der Isar, Technische Universität München, Ismaninger Street 22, 81675 München, Germany; ^2^Department of Nuclear Medicine, Klinikum rechts der Isar, Technische Universität München, Ismaninger Street 22, 81675 München, Germany

## Abstract

The secretase BACE1 is fundamentally involved in the development of cerebral amyloid pathology in Alzheimer's disease (AD). It has not been studied so far to what extent BACE1 activity in cerebrospinal fluid (CSF) mirrors in vivo amyloid load in AD. We explored associations between CSF BACE1 activity and fibrillar amyloid pathology as measured by carbon-11-labelled Pittsburgh Compound B positron emission tomography ([^11^C]PIB PET). [^11^C]PIB and CSF studies were performed in 31 patients with AD. Voxel-based linear regression analysis revealed significant associations between CSF BACE1 activity and [^11^C]PIB tracer uptake in the bilateral parahippocampal region, the thalamus, and the pons. Our study provides evidence for a brain region-specific correlation between CSF BACE1 activity and in-vivo fibrillar amyloid pathology in AD. Associations were found in areas close to the brain ventricles, which may have important implications for the use of BACE1 in CSF as a marker for AD pathology and for antiamyloid treatment monitoring.

## 1. Introduction

The cerebrospinal fluid (CSF) is in close contact with the extracellular space of the central nervous system; therefore, pathological changes such as those due to neurodegeneration are reflected in CSF and alterations of biomarkers can yield important mechanistic and diagnostic information on disease processes. In Alzheimer's disease (AD), a growing number of CSF biomarkers are studied, some of which mirror important events in the pathogenic cascade. These markers include several indicators of changes related to the amyloid cascade such as amyloid *β* 42 (A*β*42), total tau and phosphorylated tau [1]. The amyloid hypothesis of AD argues a cerebral accumulation of amyloid as primary driver of AD pathogenesis, including amyloid plaque deposition, neurofibrillary tangle formation, synapse loss, and neuronal cell death [2]. The amyloid deposits of insoluble protein material mainly contain high levels of the 40 and 42 amino acid long A*β*, which is produced through the cleavage of a membrane-bound amyloid precursor protein (APP) by the *β*- and *γ*-secretases. The cleavage of APP by the main cerebral *β*-secretase (*β*-site APP-cleaving enzyme 1, BACE1) [3] occurs at the N-terminus of the A*β*-domain and results in secreted soluble APP *β* (sAPP*β*) and a C-terminal fragment (C99) [4], which subsequently undergoes cleavage by *γ*-secretase producing A*β*. The crucial role of BACE1 in AD pathogenesis is highlighted by its increased enzyme activity and protein concentration in AD brains [5]. Several previous studies have shown that BACE1 activity can readily be detected in CSF with activity increases in mild cognitive impairment (MCI) and clinically diagnosable AD [6–8]. The relevance of BACE1 in the amyloid cascade is also underscored by its positive association with the CSF products of APP cleavage, including A*β*40 and sAPP*β* [9]. Therefore, the value of BACE1 as a clinical biomarker for AD pathology and for anti-amyloid treatment effects is currently under debate. Further in-vivo studies are urgently needed in order to explore the association between CSF BACE1 activity and amyloid pathology in AD; modern imaging techniques might play an important role in this regard since they are able to provide information about clinically suspected cerebral pathology and its spatial distribution.

Recently, the carbon-11-labeled positron emission tomography (PET) tracer Pittsburgh's Compound B ([^11^C]PIB) became available as an in-vivo imaging tool for cerebral amyloid deposits. Although [^11^C]PIB binds to several types of cerebral A*β*, including plaque, nonplaque, and vascular deposits, it has only minimal affinity to other protein aggregates such as Lewy bodies or neurofibrillary tangles [10–13]. Studies on patients with AD have demonstrated an increased tracer uptake, even in very mild clinical stages. Also, a progression of uptake with advancing clinical disease severity has been discussed [14, 15]; however, it seems that the progression of tracer uptake reaches a plateau when AD becomes clinically diagnosable [16]. The reported correlations between [^11^C]PIB tracer uptake and CSF markers related to amyloid pathology such as A*β*42 further underscore the relevance of [^11^C]PIB PET imaging as an AD biomarker [17–22].

In the present study, we quantitatively analyzed CSF BACE1 activity and [^11^C]PIB tracer uptake in 31 patients with probable AD, whose clinical diagnosis was supported by an AD typical metabolic pattern in their fluorine-18-labeled fluorodeoxyglucose ([^18^F]FDG) PET scans. Our main aim was to explore associations between [^11^C]PIB tracer uptake and BACE1 activity in CSF in order to provide evidence in support of BACE1 activity as an in-vivo biomarker for AD pathology.

## 2. Methods

### 2.1. Study Participants

Thirty-one patients with probable AD were recruited from the outpatient memory clinic of the Department of Psychiatry and Psychotherapy at the Technische Universität München. The clinical diagnosis was established by consensus of two experienced clinicians according to the NINCDS-ADRDA criteria. The clinical assessment included patient and proxy interviews, cognitive testing, physical examination, routine blood and CSF sampling, structural MRI, and [^18^F]FDG as well as [^11^C]PIB PET imaging of the brain. The psychometric workup was based on the Consortium to Establish a Registry for AD (CERAD) neuropsychological battery, which includes the Mini-Mental-State Examination (MMSE). Patients with mild to moderate dementia, who had a typical metabolic pattern for AD on [^18^F]FDG PET scans [23] were included in order to increase diagnostic accuracy. Patients were excluded from the study if they met criteria for other neurological or psychiatric disorders including Parkinson's disease, dementia with Lewy bodies, frontotemporal dementia, progressive supranuclear palsy, corticobasal degeneration, normal pressure hydrocephalus, depression, or alcohol dependence. All consecutive patients meeting the above in- and exclusion criteria were enrolled; [^11^C]PIB PET scans and BACE1 activity values were not used for diagnostic purposes. Written informed consent was obtained from all participants according to the 1975's Helsinki Declaration, and the study protocol was approved by the ethics committee of the Medical Faculty at the Technische Universität München.

### 2.2. Blood and CSF Assays

Serum and EDTA plasma samples for each subject were obtained by venous puncture for diagnostic purposes and genotyping. DNA was extracted from blood using standard procedures, and the apolipoprotein E (*APOE*) genotype was determined using polymerase chain reaction and restriction enzyme digestion according to an established standard protocol [24]. Patients were categorized as either *APOE ε*4 allele carriers or noncarriers for the present study. CSF (5–8 mL) was collected in sterile polypropylene tubes, using atraumatic cannulas placed in the L3/L4 or L4/L5 intervertebral space, and gently mixed. In the native CSF, determination of routine chemical parameters was performed. Subsequently, the CSF was centrifuged for 10 min at 4000 g, and aliquots of the remaining CSF supernatants were immediately frozen at −80°C for later determination of AD parameters. Tau and A*β*42 concentrations were measured in duplicate using enzyme-linked immunosorbent assay (Innotest Innogenetics, Zwijndrecht, Belgium) as described previously in detail [25].

BACE1 activity was measured using a time-resolved fluorescence activity assay based on SignalClimb technology (TruePoint Perkin Elmer, Turku, Finland) according to optimized manufacturer's instructions [6]. The synthetic TruePoint BACE1 substrate is a ten amino acid long peptide with a fluorescent europium (EU) chelate coupled to one end and a quencher of europium fluorescence (QSY7) coupled to the other end via lysine. The hydrolysis of the substrate's protein sequence CEVNLDAEFK by BACE1 results in a fluorescence signal proportional to the activity of BACE1. The fluorescence signal was measured at 37°C in a microplate reader using time-resolved fluorescence (FLUOstar Omega, BMG Labtech, Offenburg, Germany; excitation wavelength: 320 nm, emission wavelength: 615 nm) in black 96-well plates (Perkin Elmer, Turku, Finland) at a final volume of 27 *μ*L, including 10 *μ*L CSF, 2 *μ*L DMSO, and 15 *μ*L BACE1 substrate (0.80 nmol/mL). The continuous measurement of BACE1 was started immediately after adding the CSF sample; BACE1 activity was defined as the maximal activity within the first 30 min. Each sample was measured at least four times in order to verify reproducibility.

### 2.3. Cerebral Imaging and Image Preprocessing

Structural MRI, [^18^F]FDG PET, and [^11^C]PIB PET scans of the brain were acquired according to published standard procedures [14] within four weeks after the initial clinical assessment. SPM8 software (Wellcome Functional Imaging Laboratory, London, UK) based on Matlab v7 (The Mathworks Inc, Natick, Mass USA) was used to preprocess and analyze the [^11^C]PIB PET data. For spatial transformation of [^11^C]PIB data, the individual standardized uptake value images (40–70 min p.i.) were coregistered to the corresponding high-resolution MRI scans and then normalized to the Montreal Neurological Institute space (MNI; bic.mni.mcgill.ca) with the warping parameters of the individual's MRI to obtain images comparable between patients. In order to adjust for interindividual differences in tracer uptake, a relative measure of [^11^C]PIB was obtained by calculating a cerebral to cerebellar vermis ratio as demonstrated previously [14]; briefly, the uptake in all pixels of the subjects' [^11^C]PIB scans was divided by the individual mean value in the cerebellum. The individual normalized images were then smoothed with a Gaussian kernel of 10 × 10 × 10 mm.

### 2.4. Statistical Analysis

Patient characteristics were analyzed in the Predictive Analytics Software package (PASW) v18 (The SPSS Inc., Chicago, Ill, USA). To explore the correlation between CSF BACE1 activity and [^11^C]PIB tracer uptake, a voxel-based linear regression analysis was performed within SPM8 with tracer uptake as the dependent variable and CSF BACE1 activity as the independent variable, adjusting for factors known to impact on [^11^C]PIB uptake and amyloid pathology including *APOE* genotype [14, 26], age [27], gender [28], and interval between PET scan and CSF sampling [14]. A significance threshold of *P* < 0.001 uncorrected for multiple comparisons was applied as in several previous studies using voxel-based multiple linear regression approaches [26].

## 3. Results

Patient characteristics are shown in Table 1. The average time interval between [^11^C]PIB PET scan and lumbar puncture was 29 ± 41 days. Men and carriers of an *APOEε*4 allele were slightly overrepresented in the sample. Visual inspection of the [^18^F]FDG PET scans demonstrated a typical pattern for AD in all patients, that is, a bilateral affection of temporal and/or parietal and/or posterior cingulate cortex [23]. The visual analysis of the [^11^C]PIB scans showed an AD-typical tracer uptake as previously described in all patients, including frontal and temporoparietal cortex, precuneus, posterior cingulate gyrus, and striatum, and less retention in sensorimotor, occipital, and cerebellar cortex [26]. No patient showed a [^11^C]PIB-negative scan. The CSF concentrations of tau and A*β*42 were in the expected ranges for this type of sample [25].

**Figure 1:**
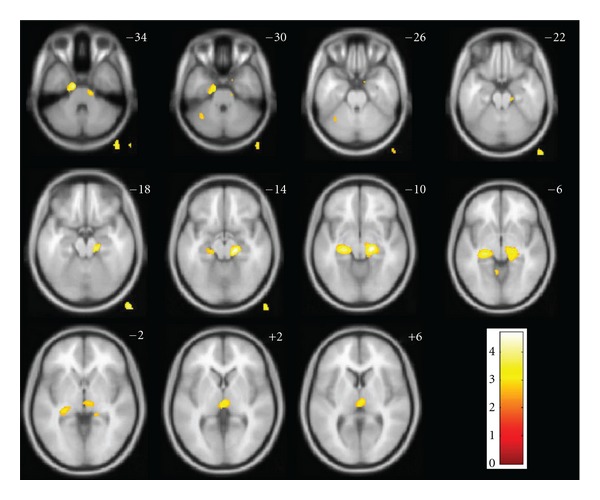
SPM8 maps of voxel-based correlations between [^11^C]PIB tracer uptake and CSF BACE1 activity. Anatomical localization as projected on axial sections of a normal T1-weighted MRI, spatially normalized into the MNI template at the given *z* coordinates in Talairach's space (*P* < 0.01 uncorrected for display purposes; voxels outside of the brain and in the cerebellum are artifacts due to this threshold); color bars represent *Z*-scores; images are displayed in neurological orientation (right side is right).

**Figure 2:**
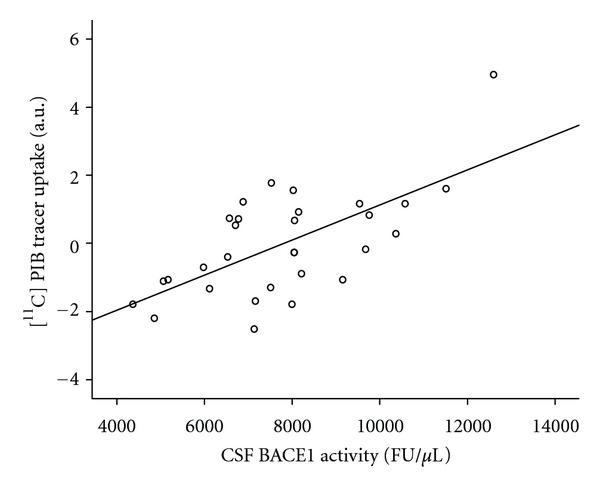
Linear regression analysis of fitted and adjusted [^11^C]PIB uptake and CSF BACE1 activity at the localization of the most significant cluster (Talairach's coordinates *x*/*y*/*z*  18/−24/−9, right parahippocampal gyrus, Brodmann's area 35), FU: fluorescence units.

The regression analysis with [^11^C]PIB tracer uptake as the dependent variable and CSF BACE1 activity as the independent variable, adjusted for covariates as described above, revealed significant positive associations in the bilateral parahippocampal region, the right thalamus, and the left pons (global maximum at MNI coordinates *x*/*y*/*z* 18/−24/−9, right parahippocampal gyrus, Brodmann area 35, *P* < 0.001 uncorrected). No other brain region showed a significant correlation between BACE1 activity in CSF and [^11^C]PIB tracer uptake (Table 2, Figures 1 and 2).

## 4. Discussion

APP cleavage by BACE1 is the rate-limiting step in the production of A*β* and the activity of BACE1 in CSF can readily be measured [29]; thus, CSF-based detection of BACE1 activity might be valuable in the early detection, differential diagnosis, and anti-amyloid treatment monitoring in AD. Our results not only confirm findings of previous studies that suggest an association between BACE1 activity and in-vivo amyloid pathology [5, 9] but also extend these earlier investigations by presenting evidence for a region-specific pattern of this relation, reporting a significant positive correlation between BACE1 activity in CSF and [^11^C]PIB tracer uptake in the parahippocampal region, the thalamus, and the pons.

Increased BACE1 expression, concentration, and activity in and around senile plaques have been reported in several studies in cognitively healthy elderly individuals and patients with AD [5, 30, 31]. BACE1 activity increases, and correlations with amyloid pathology were most consistently found not only in AD-vulnerable brain regions such as the temporal cortex, the hippocampal region, as well as the prefrontal cortex [32] but also in some less vulnerable structures in the diencephalon and brain stem including the thalamus and the pons [33, 34]. Furthermore, a coexpression of BACE1 and APP, which is a prerequisite for A*β* production, in the hippocampal region has been reported in APP/BACE1 transgenic mice [35–37]. The findings of these postmortem and animal model studies are corroborated by our in-vivo findings; however, associations between BACE1 activity in lumbar CSF and amyloid load in [^11^C]PIB PET scans were not found in some of the previously reported brain regions including the prefrontal cortex [31].

In line with our recent finding that the correlation between CSF A*β*42 and [^11^C]PIB tracer uptake is strongest in brain regions close to the ventricles [20], our present results may indicate that BACE1 activity measured in lumbar CSF primarily reflects amyloid pathology in structures bordering the ventricular system. This observation can possibly be explained by the fact that CSF is drained from the ventricles into the spinal space before a small part of it reaches cortical areas such as prefrontal cortex [38] as well as the fact that proteins from ventricle remote regions are drained via other ways including the perivascular interstitial fluid channels following the cortical arteries [39]. Therefore, BACE1 activity measured in lumbar CSF may predominantly correspond to the enzymatic activity located in specific brain structures. As an alternative explanation, brain regional BACE1 activity differences might actually exist in patients with clinically diagnosable AD, and the plateau phase of [^11^C]PIB uptake increase in this disease stage might further restrict the association to certain brain areas.

Some limitations of our study have to be mentioned. The patients were recruited from a specialized university center and may, therefore, not truly represent the whole population with AD. Furthermore, a modest number of patients were included and replication in larger samples would be helpful. An additional concern is the lack of pathologic confirmation of AD. However, the validity of present clinical diagnostic criteria compared with autopsy diagnoses has been reported to be very good in study cohorts recruited at specialized centers [40]; moreover, only patients with a typical metabolic pattern for AD on [^18^F]FDG PET scans were included. Finally, although our cross-sectional study demonstrated a correlation between BACE1 activity and in vivo amyloid load, longitudinal studies are necessary to determine the stability or variability of this association over time.

To conclude, our results point to an association between BACE1 activity in lumbar CSF and in-vivo fibrillar amyloid pathology in regions adjacent to the brain ventricular system. Thus, our data implicate that CSF BACE1 activity reflects AD pathology in particular brain regions, whereas [^11^C]PIB tracer uptake may map the entire cerebral amyloid load. This observation suggests that BACE1 activity in CSF can only be considered a peripheral measure for AD pathology in relation to certain brain regions. Our results support the notion that CSF analysis may represent an important tool for the detection of ongoing intracerebral pathology, but complementation with imaging procedures may allow the assessment of actual presence, quantity, and regional distribution of pathology in the brain. It is also important to note that the decision for or against CSF and imaging biomarkers in clinical routine and treatment trials will also depend on the willingness among clinicians to perform and the willingness of patients to undergo lumbar puncture on the one hand and access to cyclotrons and PET scanners as well as financial considerations on the other hand. Our findings encourage further studies in order to explore the value of BACE1 activity in CSF as a reliable, affordable, and easily accessible AD biomarker. However, our results also narrow the possible applications of CSF BACE1 activity due to its selective correlation with brain regional amyloid pathology.

## Figures and Tables

**Table 1:** Description of the study sample.

*N*	31
Male : female	19 : 12
Age, years*	65.8 (7.86)
MMSE*	23.9 (4.41)
CSF BACE1 activity, FU/*μ*l*	7793 (2003)
*APOE ε*4+ : *ε*4−	17 : 14
CSF tau (ng/L)	635.58 (310.39)
CSF A*β*42 (ng/L)	523.74 (116.79)

*mean (SD), FU: fluorescence units.

**Table 2:** Peak correlations between [^11^C]PIB tracer uptake and CSF BACE1 activity.

Region	*x*	*y*	*z*	*Z*-score	Cluster
Parahippocampal gyrus (r), BA 35	18	−24	−9	3.91	733
Parahippocampal gyrus (l), BA 28	−24	−24	−6	3.58	372
Parahippocampal gyrus (l), BA 35	−14	−13	−28	3.51	235
Thalamus (r)	2	−19	3	3.02	733
Pons (r)	8	−20	−38	3.12	495

Significant brain clusters were labeled in Talairach daemon software (talairach.org/Daemon.html) after conversion of MNI (bic.mni.mcgill.ca) to Talairach's coordinates using the Matlab function mni2tal (http://imaging.mrc-cbu.cam.ac.uk/imaging/MniTalairach); brain regions are indicated by Talairach's coordinates; cluster: extent of contiguous voxels within the cluster; r: right, l: left; BA: Brodmann's area.
